# Supplementary figures and images for: The mre11 A470 alleles influence the hereditability and the segregation of telosomes in Saccharomyces cerevisiae
Source: PLoS One. 2017 Sep 8;12(9):e0183549. doi: 10.1371/journal.pone.0183549 (PMC5590830; doi:10.1371/journal.pone.0183549)

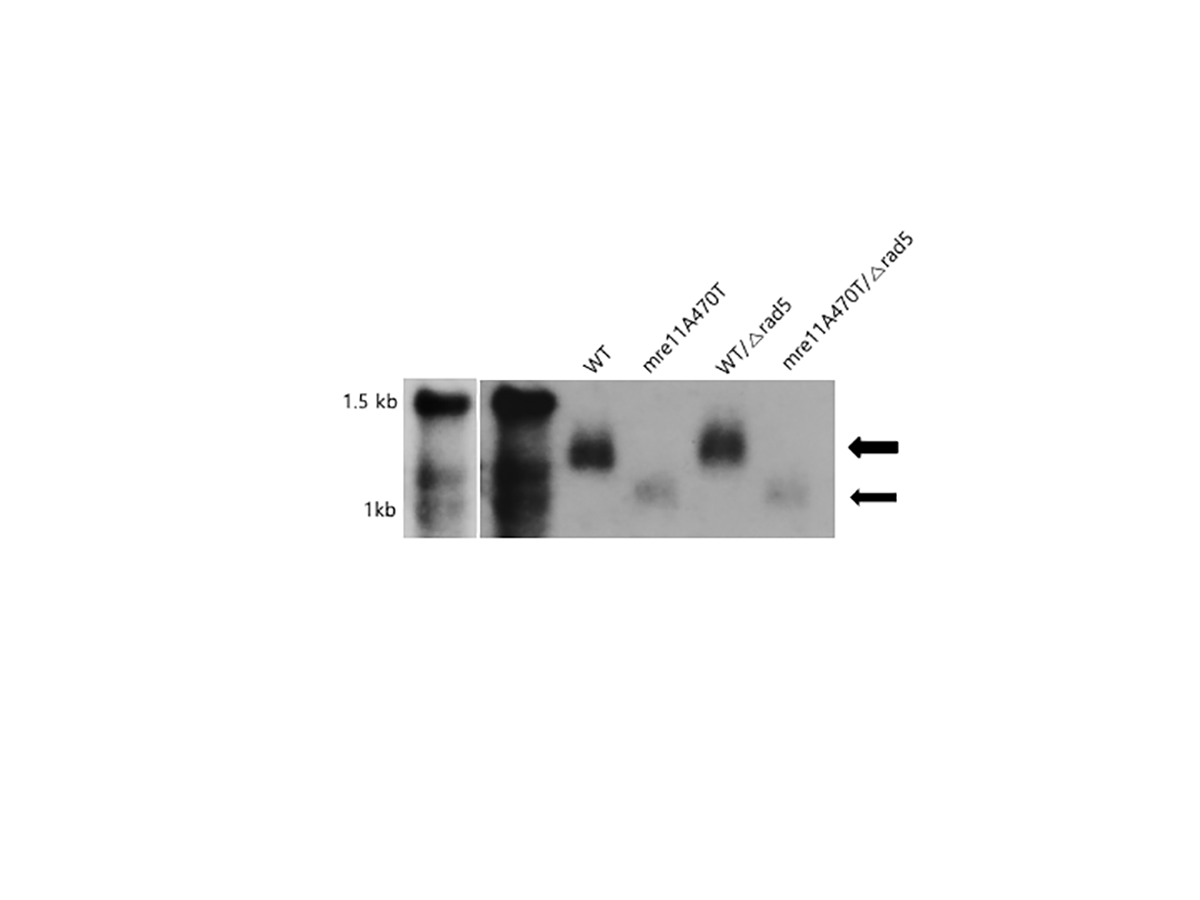

Supplement: S1 Fig — XhoI digestion of DNA from the following genotypes were tested: Lane1, MRE11 RAD5; lane2, mre11A470T RAD5; lane3, MRE11rad5△; and lane 4 is mre11A470T rad5△ strains. The rad5△ deletion did not give rise to a telomere size change. The mre11A470T rad5Δ double mutant has the same short telomere phenotype and heritability as mre11A470T cells. (TIF) [file pone.0183549.s001.tif]

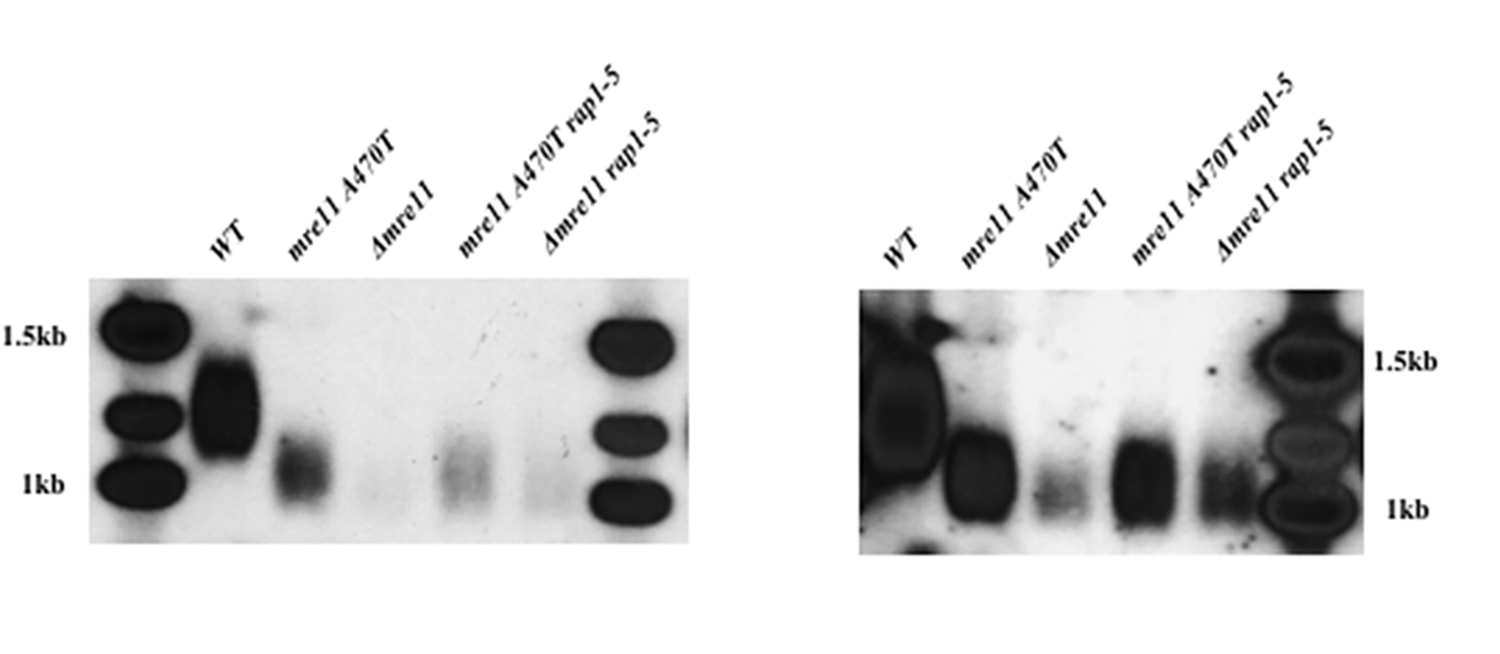

Supplement: S2 Fig — We extracted DNA from WT, mre11A470T, mre11Δ, mre11A470T rap1-5, and mre11Δ rap1-5 chromatin. The DNA was digested with XhoI and subjected to Southern blot analysis using the A750 probe. Two exposures are shown to visualize all fragments. (TIF) [file pone.0183549.s002.tif]

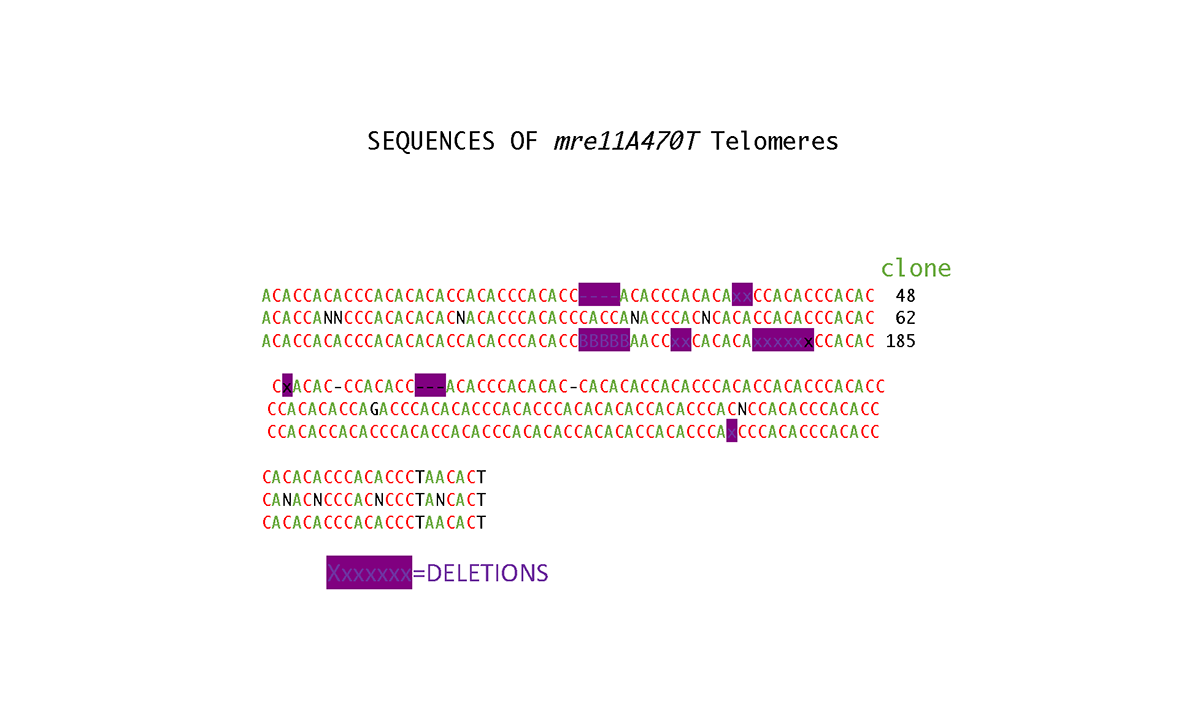

Supplement: S3 Fig — We sequenced multiple X- class telomeres from independent clones of wild-type or mre11A470T cells using the C-tail method [51] with the X-class primers adjacent to a telomere tract. The X-class telomeres represent a group of 13 telomeres that have identical junctions with the telomere. S3A: Sequence of three clones of a mre11A470T was conducted on independent telomeres derived from a single source. The color code is present simply for convenience and is not meant to indicate causality. (TIF) [file pone.0183549.s003.tif]

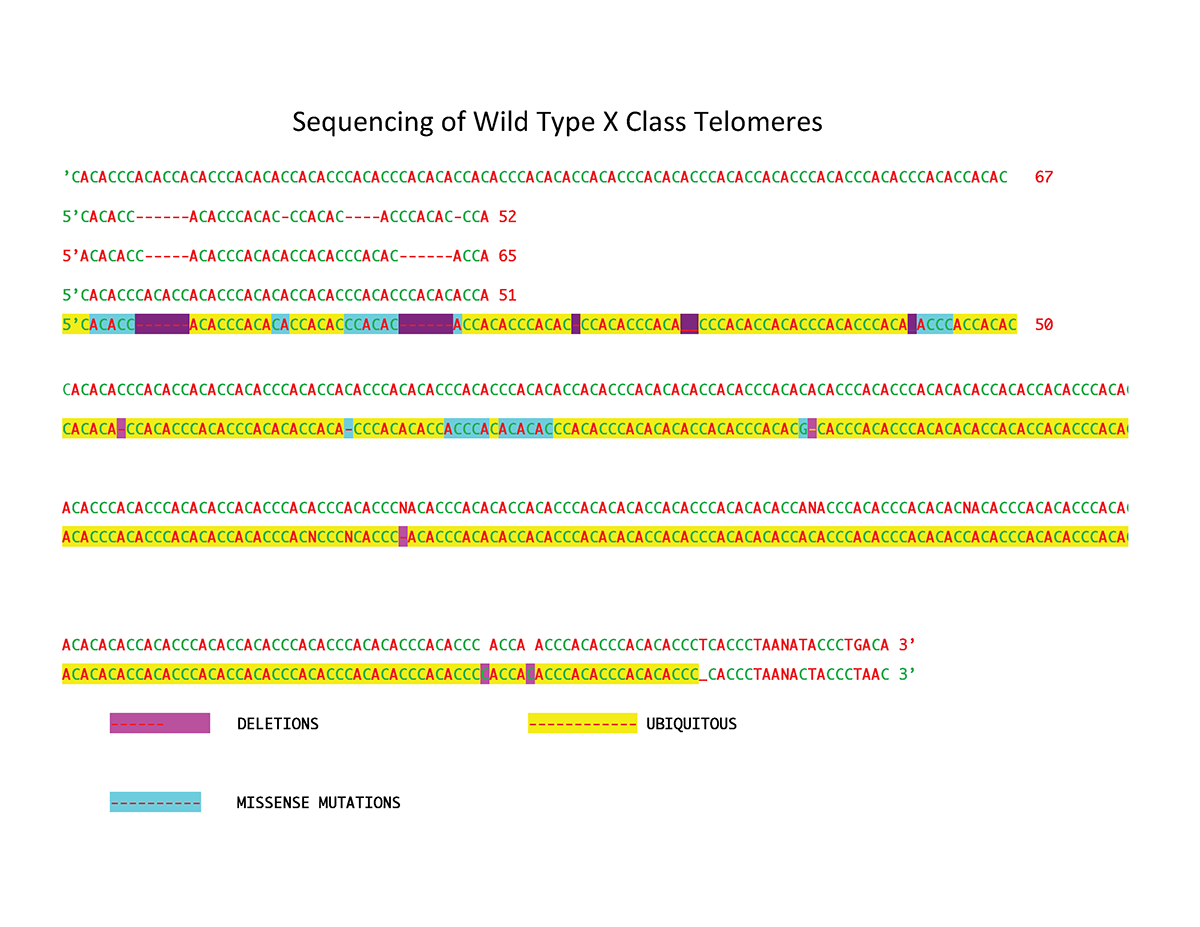

Supplement: S4 Fig — The top sequences run from the telomere proximal to distal direction. The color code denotes the type of misalignment or gap among different telomeres even when they were in the minority. The comparisons are not used to determine causal relationships, simply the type of events that are occurring. (TIF) [file pone.0183549.s004.tif]

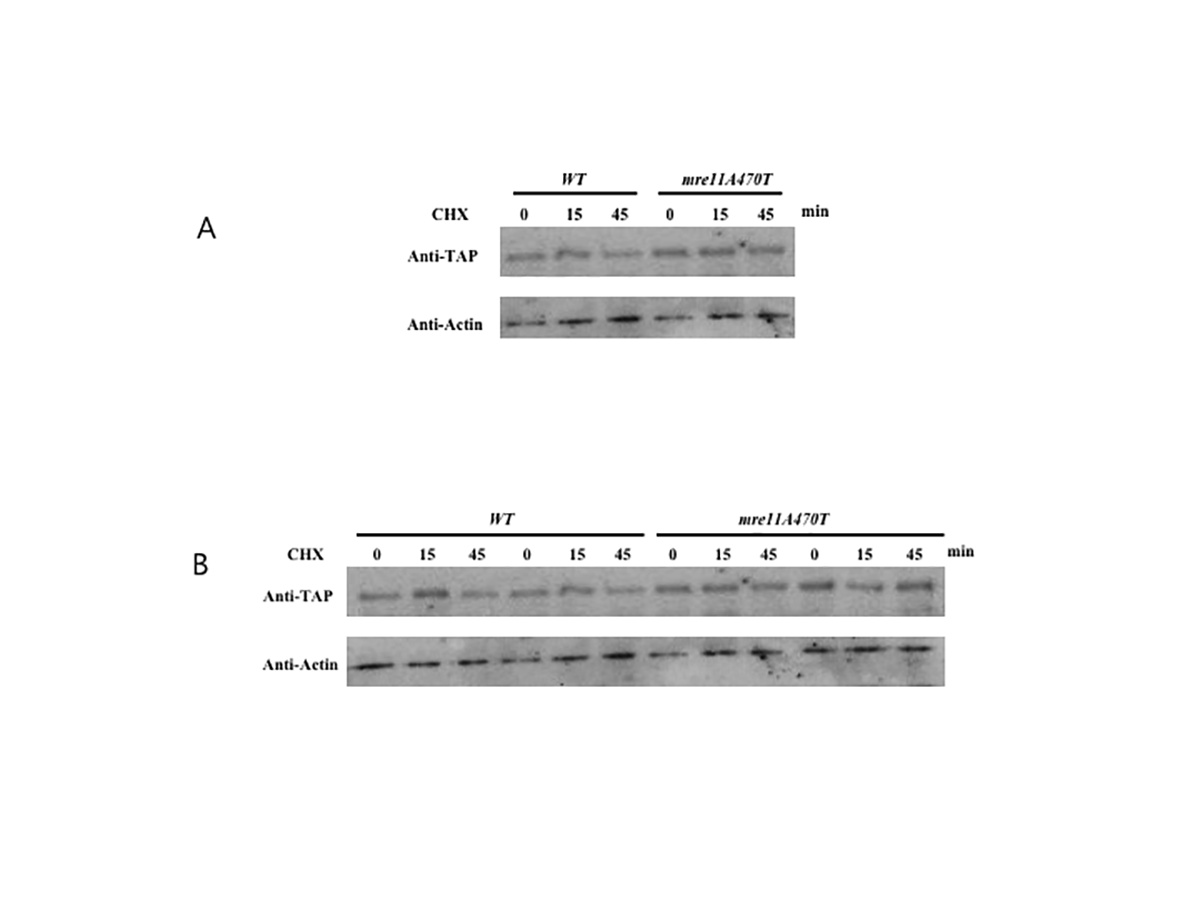

Supplement: S5 Fig — [A] Stability assay for Mre11-Tap and Mre11A470T-A portion of the CHX stability assay of strains containing a locus at the genomic site and TAP-tagged derivatives [Mre11-TAP and Mre11A470PT-TAP}. Westerns were probed with anti-TAP antibody antibodies as described in part B. The protein migrates more slowly due to the TAP tag. Size markers are provided on the left. The blot was stripped and re-probed with anti-acting antibody. [B] CHX protein stability assay was performed in MRE11 and mre11A470T strains. Cells were grown in YPD to mid-log phase, and 100 μg of cycloheximide was added. Cells were incubated and collected at designated time point. The proteins were prepared, and Mre11 antibody was used to detect Mre11 and Mre11A470T protein. Mre11A470T has the same protein stability as Mre11. Actin was observed after stripping the blot and probing with an anti-actin antibody. (TIF) [file pone.0183549.s005.tif]

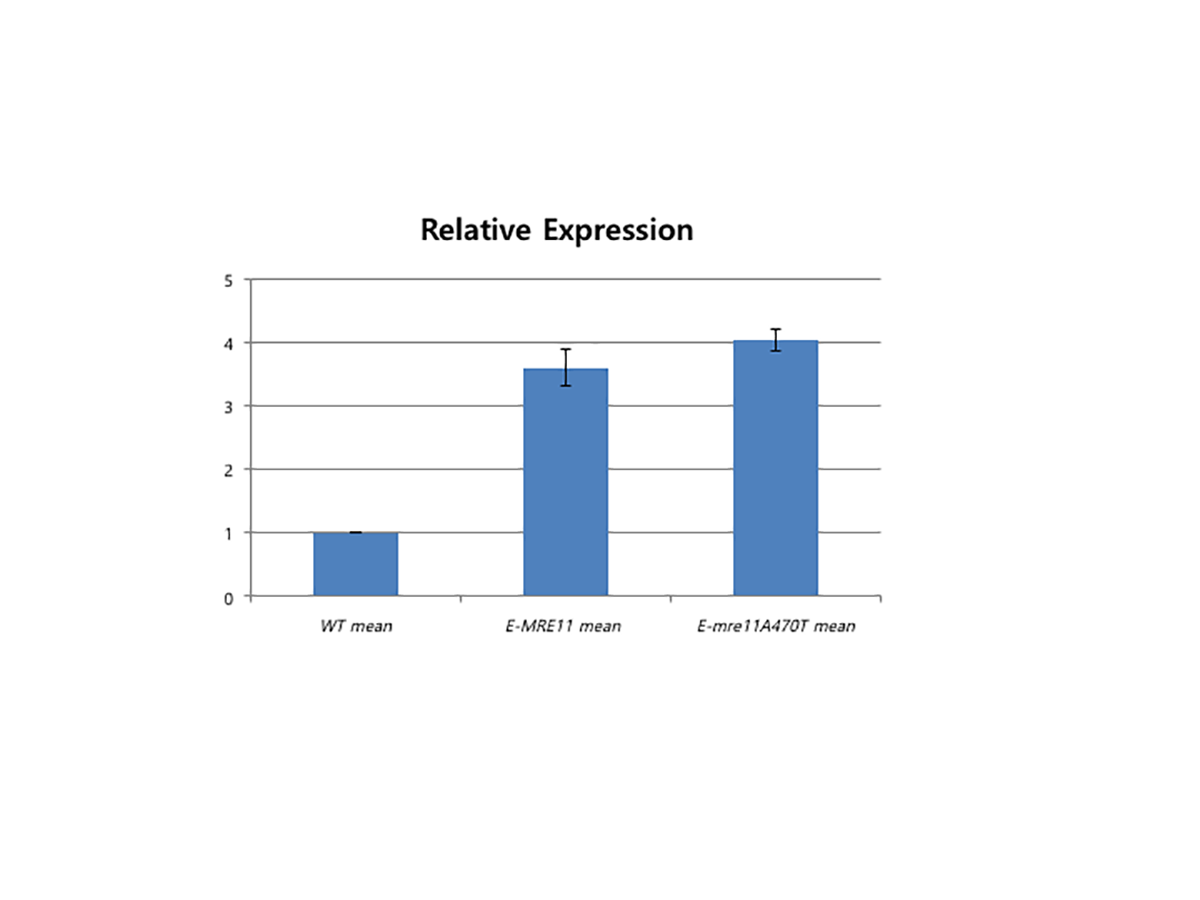

Supplement: S6 Fig — To test the transcriptional expression level of overall Mre11 in strains carrying two loci [genomic and ectopic], real time-qPCR of the reverse transcribed RNA was conducted as described in Materials and Methods. The first, second, and third bars refer to WT [g-MRE11] [as a control], e-MRE11 g-MRE11 and e- mre11A470T g-MRE11 strains, respectively. The expression of both e-MRE11 g-MTE11 and e-mre11A470T g-MRE11 were three- to four-fold greater than strains having only a single genomic locus. The Y axis represents the fold increase in transcripts relative to wild type. (TIF) [file pone.0183549.s006.tif]
